# Perception of the Ethical Climate Among Hospital Employees in a Public Healthcare System: A Qualitative Study at the University Hospital of Split, Croatia

**DOI:** 10.3390/healthcare14060735

**Published:** 2026-03-13

**Authors:** Zrinka Hrgović, Luka Ursić, Jure Krstulović, Ljubo Znaor, Ana Marušić

**Affiliations:** 1 Department of Family Medicine, Health Center of Split-Dalmatia County, Kavanjinova 2, 21000 Split, Croatia; 2 University Postgraduate Doctoral Study Program Translational Research in Biomedicine, University of Split School of Medicine, Šoltanska 2A, 21000 Split, Croatia; jkrstulovic@kbsplit.hr; 3 Department of Research in Biomedicine and Health, Centre for Evidence-Based Medicine, University of Split School of Medicine, Šoltanska 2A, 21000 Split, Croatia; luka.ursic@mefst.hr (L.U.); ana.marusic@mefst.hr (A.M.); 4 Department of Health Care Quality, University Hospital of Split, Spinčićeva 1, 21000 Split, Croatia; 5 Department of Surgery, University Hospital of Split, Spinčićeva 1, 21000 Split, Croatia; 6 Department of Ophthalmology, University Hospital of Split, Spinčićeva 1, 21000 Split, Croatia; lznaor@kbsplit.hr; 7 Department of Ophthalmology, University of Split School of Medicine, Šoltanska 2A, 21000 Split, Croatia; 8 Department of Research, University Hospital of Split, Spinčićeva 1, 21000 Split, Croatia

**Keywords:** ethical climate, healthcare professionals, focus group interviews, Croatia, Hospital Ethics Committee, medical ethics

## Abstract

**Background/Objectives**: The ethical climate in a healthcare institution encompasses the shared perceptions of how ethical issues are managed in everyday practice. Our prior survey at the University Hospital of Split, Croatia, showed a simultaneous predominance of the “Rules” and “Laws and professional codes” ethical climates. Building on these findings, we explored how these climates manifest in everyday practice, how they align with staff values and guide their ethical decision-making, and how they are shaped by external factors. **Methods**: We conducted seven focus groups with 31 participants: nurses, residents, specialists, and members of the Hospital Ethics Committee (HEC). We identified patterns in the data using Graneheim and Lundman’s qualitative content analysis. **Results**: Three themes emerged from our analysis. We observed that the ethical climate was shaped predominantly by healthcare professionals themselves based on shared professional values and informal norms, rather than explicit institutional rules. Nurses, positioned as frontline workers, felt particularly exposed to ethical dilemmas, reporting perceived subordination to physicians, increased pressures from patients, and vulnerability in ethically ambiguous situations. The participants generally believed that institutional leadership insufficiently utilised existing tools, bodies, and mechanisms to support ethical behaviour and sanction misdemeanors, resulting in gaps in human resource management, a lack of practical protocols, and a weak HEC. **Conclusions**: To strengthen the ethical climate, institutional leadership should provide clear and practical guidelines, effectively utilise regulating bodies and support services, establish dedicated mechanisms to support nurses, and consistently enforce sanctions for unethical behaviour.

## 1. Introduction

Clinicians face moral and ethical dilemmas in their practice every day, both in interactions with patients and with colleagues [1]. These dilemmas, spanning from diagnosis and treatment to administrative difficulties, are frequently challenging to discuss and resolve as a team [2], since management, doctors, and nurses need to align on the ideal approach for tackling the matter at hand [3]. The “ethical climate” becomes important in this context, as it refers to the shared perceptions of what is ethically correct behaviour and how ethical issues should be handled within an organisation [4]. According to Victor and Cullen [5], it comprises five subtypes, the “Instrumental”, “Caring”, “Independence”, “Laws and codes”, and “Rules” climates, each reflecting different norms and values that influence the conduct of employees at an organisation.

In an “Instrumental” climate, behaviour is guided by one’s own or a company’s interest. A “Caring” climate is based on concern for others, which is embedded in an organisation’s policies and practices. An “Independence” climate indicates that individuals follow their own personal and moral beliefs, despite external mediators. The “Laws and codes” climate is one in which an organisation supports principled decision-making based on external legal and professional codes of conduct. A “Rules”-oriented climate is expected to be guided strictly by company rules and procedures [4,6]. The latter two climates share a principal construct, and some studies have shown that, in cases where both are present, employees conduct themselves by established rules and legal regulations [7,8]. For this reason, researchers grouped them under the umbrella term “normative ethical climate” [7]. The perception of these two positive ethical climates is highly correlated with organisational citizenship behaviour, which is characteristic of employees who will exceed their official job duties to actively support their organisation [9,10]. Furthermore, the presence of a positive ethical climate within an organisation has been recognised as crucial, since it has been linked to better nurses’ task performance [11], job satisfaction, organisational support, and commitment [12,13], as well as clinicians’ effective teamwork [14].

In the context of healthcare, the ethical climate reflects how workers view the handling of ethical challenges in their workplace [15]. In practice, they often have to follow a specific set of rules, even if they go against their own principles, which may lead to moral distress [16]. They are also always required to prioritise a patient’s well-being through a methodical, ethically sound decision-making process [17]. However, when ethical conflicts arise and when support for their resolution is lacking, their ability to provide ethically good care or their perception of doing “ethically good” work may be compromised [18].

Research on the ethical climate in Croatia remains scarce, as studies have mainly focused on higher education institutions [19,20,21]. For example, two surveys identified “Company rules and procedures” and “Laws and codes” as the predominant ethical climates at three schools at the University of Split, Croatia [19,20]. The third, interview-based study at two of these three schools highlighted how, in such contexts, institutional leaderships can shape how the climate develops and, consequently, how employee satisfaction and performance are affected [21]. To our knowledge, no such studies have been conducted at healthcare institution in Croatia, where such data could be used by decision-makers to improve the climate among staff, which could lead to better care for patients downstream.

To address this gap, we performed a survey at the University Hospital of Split, a publicly funded hospital in Croatia [22], in which we identified the “Rules” and “Laws and professional codes”, jointly considered the normative ethical climate, as predominant among healthcare staff. This implies that employees tend to be guided by organisational rules and shared norms and values, rather than by individuals’ moral principles. However, although such surveys can identify prevalent ethical climate types, they cannot provide insight into how they manifest in everyday clinical practice. To contextualise these findings, we conducted a series of focus groups within the University Hospital of Split to explore how healthcare professionals perceive the influence of these two climates on their approaches to resolving ethical dilemmas, how these normative ethical climates interact with their personal values, and which factors may affect the ethical climate. Additionally, we aimed to explore whether the Hospital’s ethical code, which is currently under development, could support healthcare professionals’ daily work.

## 2. Materials and Methods

We conducted a focus group-based study among nurses, residents, specialists, and members of the Hospital Ethics Committee (HEC) at the University Hospital of Split to explore their perceptions of the Hospital’s ethical climate and the factors affecting it. We reported our findings per the Consolidated Criteria for Reporting Qualitative Research (COREQ) guidelines [23] (Supplementary Table S1).

### 2.1. Study Setting

The University Hospital of Split is the second largest hospital in Croatia, providing specialised, tertiary-level healthcare services to a catchment area of several southern counties in Croatia, with a population of almost 800 thousand. It provides over 450,000 inpatient days to more than 50,000 patients and approximately 2.5 million outpatient procedures to more than 600,000 individuals each year [24]. It is also affiliated with the University of Split School of Medicine through teaching and research activities.

### 2.2. Sampling

We purposefully sampled participants from each of the four study subgroups: nurses, residents, specialists, and members of the HEC. Specifically, the research team members working at the Hospital (JK and LjZ) engaged staff directly at their workplace and asked them to participate, or to recommend someone who might be interested in participating. We set no eligibility criteria in terms of sex, age, or years of service, and only defined that the person had to be a nurse, resident, or specialist working at the Hospital. We otherwise enrolled all members of the HEC.

One author (JK) approached potential participants in person and invited them to participate in the study after providing information on its methodology and goals and detailing how their anonymity would be maintained and their data protected. Participants were informed that participation was voluntary and that they could withdraw at any stage without consequences.

### 2.3. Reflexivity

The research team included five members from Croatia: three men (JK, LU, LjZ) and two women (ZH, AM). JK is employed at the Department of Health Care Quality, LjZ at the Department of Ophthalmology, while ZH is employed at the Department of Family Medicine at the Health Center of Split. LU, LjZ, and AM are employed at the University of Split School of Medicine, which is affiliated with the Hospital, while both LjZ and AM also work part-time at the Hospital’s Science Department. Due to JK’s and LjZ’s positions in the Hospital, their personal relationships with some of the participants, and the fact that LjZ heads the HEC, they likely had their own interests and perspectives in the participants’ perceptions of the ethical climate. We mitigated this by having a third person (ZH) conduct the interviews and the qualitative analysis. She had spent some time at the Hospital during her clinical rotations and learned how the institution operated on a daily basis, and even knew some of the participants, but was not employed at the institution and had no perceived gains from the findings of the focus groups. To further mitigate bias, we engaged an independent observer (LU) experienced in qualitative methodology to monitor each focus group and participate in the qualitative analysis.

### 2.4. Context, Data Collection, and Transcription Process

The focus groups were held in person between 15 May 2024 and 27 June 2024 at the University Hospital of Split, where they were recorded using an external audio device (Olympus WS-853 with Olympus ME33 boundary microphones; Olympus, Tokio, Japan). They lasted approximately 34–56 min, were conducted in Croatian, and were moderated by ZH, with LU acting as an independent observer and taking field notes for later analysis. The participants were presented with an informed consent form prior to the focus groups, after which they filled out a short questionnaire with their demographic data (sex and level of education).

We used a pre-developed topic guide to keep the discussions focused on the study aims (Supplementary File S1). The topic guide remained unchanged throughout the process, except for the focus group with members of the HEC, where it was expanded to capture their role in developing the Hospital’s ethical code, their awareness of and training in ethical standards in healthcare, and their activities in overseeing the reporting and management of ethical dilemmas within the institution. We transcribed the focus group audio recordings verbatim, with ZH transcribing them manually and JK conducting a quality check. We then anonymised and de-identified the transcripts, which were held securely alongside the recordings on the researchers’ password-protected devices. We did not return the transcripts, field notes, or results to the participants.

### 2.5. Analysis

The focus group interviews were analysed through Graneheim and Lundman’s qualitative content analysis [25]. Initially, one researcher (ZH) read the transcripts multiple times and contextualised them with the focus group notes. She then identified meaning units, condensed them, and subsequently collated them into codes. These codes were then reviewed, relabeled, and organised into subthemes and themes. Another researcher (LU) independently performed the same procedure. Both researchers then compared and discussed their interpretations, after which they finally defined the subthemes and themes and their relationships, setting them into a thematic map.

We did not consider the concept of data saturation, as we wanted to explore how the four subgroups differed in their perspectives on the ethical climate, while rooting our investigation in the findings of our survey. We grounded this methodological choice on two reasons related to the concept of information power [26]. First, we had a narrow study aim, where we explicitly wanted to explore how the two normative ethical climates identified in our survey manifest among the Hospital staff. Our goal was therefore to deepen our existing empirical and theoretical insight, rather than to gain a full understanding of all aspects of the ethical climate in the Hospital and propose a novel theory. Relatedly, we theorised that saturation in the context of our single center could only be achieved if we obtained respondents from all Hospital departments, as experiences with the ethical climate could differ between these groups (e.g., nurses at different departments). This would have been unfeasible, however, given that coordinating the already overburdened healthcare professionals across departments and shifts would have been difficult. We thus prioritised the quality of dialogue, as our research team members (including the interviewer) worked in the Hospital alongside the staff as medical professionals and shared a common background, facilitating focused interaction that provided rich, aim-related data.

In view of data triangulation, our analysis was guided by the inclusion of several subgroups within the Hospital, our knowledge that the HEC was working on the Hospital’s ethical code as the focus groups were being conducted, and by the findings of our previous survey. We developed the meaning units, codes, sub-categories/categories, and themes in Croatian and translated them alongside select quotes for presentation in this article. We conducted the coding in a Microsoft Excel spreadsheet.

### 2.6. Ethics Considerations

The University Hospital of Split Ethics Committee (Document Class: 500-03/23-01/243, Reg. No. 2181-147/01/06/LJ.Z.-23-02 on 27 November 2023) provided ethical approval for this study. All participants were asked to provide written informed consent after being informed of the study’s purpose and methodology, that the discussions would be audio recorded, and that all data would be stored securely and accessible only to the research team. They were also informed that only selected, anonymised, de-identified, and translated quotations would be used in the manuscript and that the analysis would be conducted at the group rather than the individual level. Also, they were informed about data retention and how to contact the data protection officer if they wished to withdraw their personal data.

## 3. Results

We conducted seven focus groups—three with nurses, one with residents, two with specialists, and one with the HEC. There were thirty-one participants in total. The nurses were predominantly female, the residents and specialists had an equal representation of men and women, and all members of the HEC were female. The participants were highly educated overall, with almost all nurses holding at least a bachelor’s degree (Table 1).

Three themes and six subthemes emerged from our analysis (Supplementary Figure S1). We present them below alongside participant quotes, denoted in the text with “Q#” and summarised in tabular format at the end of each theme.

The first theme contextualised the results of our survey and showed how the two normative ethical climates manifested in practice and how they aligned with the values of the Hospital staff. It highlighted that the simultaneous predominance of the “Company rules” and “Laws and professional codes” climates reflected the institution’s implicit expectations of the staff. These expectations were incidentally aligned with their professional and personal values, meaning that the staff did not follow some explicit institutional codes and regulations, but rather operated by their professional codes and obligations. The second and third theme both reflected the factors that affected the ethical climate at the Hospital, and relatedly explored whether the ethical code under development at the time of the study could help improve it in any way. Specifically, the second theme focused on nurses, who perceived themselves to be more vulnerable to ethical dilemmas than other groups, as they were “frontline” workers exposed to internal pressures (e.g., mistreatment from MDs) and external factors (e.g., threats from patients and their families). The last theme, partially driven by the aforementioned absence of concrete rules, protocols, and codes from the Hospital administration, concerned the staff’s perceived lack of authority from the Hospital leadership, manifested through its mismanagement of human resources and inadequate utilisation of top-down support, punitive measures, and existing tools and bodies (e.g., psychosocial support or ethics committee). Despite this, the staff still wanted the Hospital administration to exercise its authority through these means, for the betterment of the ethical climate and all staff.

### 3.1. Theme 1: The Ethical Climate Is Formed by People and Teams Based on Shared Professional Values

This theme demonstrates that the Hospital set no legal or behavioural framework for the staff per se, but rather implicitly expected them to conduct themselves according to their professional codes. The employees consequently formed the climate themselves based on their shared backgrounds, with “newcomers” to each team and department having to adapt to its specificities. They also resolved ethical dilemmas either by themselves or through team discussions (collaboratively or hierarchically, i.e., by referring to their direct superiors), which was reflective of the same phenomenon.

#### 3.1.1. Subtheme 1: Predominance of “Company Rules” Climate Is Driven by Its Alignment with “Professional Codes”, Not by the Rules Themselves

The finding of our survey—that the “Company rules” and Professional codes” climates coexisted at the Hospital—likely reflected the alignment of the former with the latter, meaning that the institutional rules were, in practice, based on the staff’s professional codes (quote (Q)1). In this sense, our participants noted that they acted “as the institution expected them to because [they] were professionals” (nurse, FG1, S2). However, the Hospital seemingly provided no top-down “formal guidelines” in reality, meaning that the rules followed by the Hospital staff did not originate from an external source, but rather came “intrinsically, from the people” (resident, F4, S4). This seemed to be true for all professional subgroups, with one nurse observing that the rules they follow “came from their professionalism and their duty as medical nurses” (nurse, F1, S2). These rules, therefore, were actually rooted in their professional values, which were instilled into them through education by default (Q2). On the whole, this indicated that the institution did not directly affect the climate through rules or regulations, but rather through implicit expectations or the perception thereof from its staff. While this meant that the climate depended on individuals and could “be brought down by one person on their own” (nurse, F2, S1), it also meant that it was formed through communal, shared values (Q3).

It would, therefore, seem that the institution did not play an active role in how the ethical climate in the Hospital was formed. It set no specific guidelines—i.e., no “Company rules”—but only implicitly expected its staff to follow the professional codes they acquired during education. The staff, meanwhile, followed the codes of their profession automatically, as they all accepted them when they decided to practice health care in the first place.

#### 3.1.2. Subtheme 2: Ethically Dubious Situations Are Resolved Hierarchically Within a Team Based on Professional Standards and Expectations

Across all subgroups, the approach to resolving ethical conflicts reflected the “traditional” hierarchy of a medical team, rather than a dependence on institutional protocols or guidelines. Nurses encountering ethical dilemmas, for example, reacted “professionally, first and foremost” (nurse, F1, S1) and in the framework of “their professional competencies” (nurse, F1, S2). If they could not resolve an ethical issue themselves, they discussed solutions “mainly within their department” (nurse, F2, S2), after which they turned to the head nurse or MD, without involving the institution (Q4). The same was true for residents, who “presented cases of conflict in morning meetings, with the leading MD having the final say” (resident, F4, S5). In time-constrained dilemmas, such as those encountered at the emergency ward, they “decided what [they] think is best, but consulted the senior MDs as soon as time allows” (resident, F4, S2). This had as much to do with the hierarchy as it did with the legal and ethical responsibility carried by senior MDs and not the residents (Q5)—a fact that allowed the latter to “not stand behind the decision [of the former] if it contradicted their beliefs” (resident, F4, S6). At the top of this within-team hierarchy were specialists, who discussed any dilemmas among their peers or with more experienced colleagues, but still held that the final responsibility for decisions in such cases—both legally and ethically—remained with themselves (Q6) (Table 2).

As in forming the ethical climate, the staff did not rely on the institution when tackling ethical challenges. Instead, they took on a stratified approach, first attempting to tackle them on their own, then consulting their peers, and finally referring to a higher authority. This process was locked in within their team or department, with the “higher authority” always being a direct supervisor, senior nurse, or senior MD, rather than a dedicated body within the Hospital.

### 3.2. Theme 2: As “Frontline Workers”, Nurses Are the Most Sensitive to the Ethical Climate and Its Shifts

Subjectively, the nurses reported feeling “subordinate” to MDs within their medical teams, as they believed they had to take on work within the MDs’ purview. This placed them in an ethically precarious position, where they thought they could be held accountable for errors in performing tasks outside of their domain, despite not being culpable in a legal sense. They also felt that the Hospital leadership preferentially protected MDs and “privileged” patients against their interests or needs. Yet some nurses perceived that newer generations of healthcare staff were more respectful towards them in this sense, alleviating their feeling of professional “inferiority” and allowing them to voice their concerns about ethical problems. Others believed the contrary, observing that younger staff members did not recognise the “hierarchy” within the Hospital and that they lacked respect for patients and vice versa. In doing so, they implicitly reaffirmed the “old hierarchy” in which they felt subordinate to MDs, suggesting it to be something positive, “expected”, or “natural” from their perspective, even though it diminished their own rights.

#### 3.2.1. Subtheme 1: Nurses Feel “Subordinated” to MDs and Experience More Confrontations with Patients than Other Staff Members

The nurses reported running into points of friction with MDs in their daily work and in handling ethical dilemmas specifically (Q7, Q8). Some, for example, said they felt they had to perform duties that were otherwise supposed to be done by MDs. Others reported handling ethically dubious cases on their own because “they could not talk them out with the MDs” (nurse, F3, S3). These subjective feelings were exacerbated by the ethical challenges nurses faced when having to inform patients on surgical procedures—a task “that should be done by MDs, but isn’t” (nurse, F3, S5). Taken together, this left them feeling vulnerable to “being called out [by patients for malpractice] for something that isn’t their responsibility” (nurse, F3, S5).

The nurses also felt that, in cases of malpractices or infringements of ethical codes, the Hospital administration would protect MDs more than other staff members, granting them a privileged status, as well as that they seemed to be less protected than MDs in the media when it came to publicised, controversial cases (Q9). They likewise thought that the MDs generally enjoyed higher respect from patients (Q10), with one nurse citing a case where a patient threatened to “throw her into the sea if his operation got delayed, but calmed down and waited when the MD came” (F1, S4). Interestingly, this was also observed by one specialist, who stated that “we [MDs] wear a stethoscope, so people perceive us differently. They’re usually nice and polite, and don’t question us much […]” (specialist, F5, S1).

Taken as a whole, these factors made nurses feel as if they were in a precarious, sensitive position, thrust “between the patient and the MD, exposed on all sides” (nurse, F3, S2). The frustration with the MDs also led them to implicitly foster an “us vs. them” mentality within their team, as they expressed a need for the Hospital to provide them with uniforms that would “indicate who the MD, who the nurse, and who the head nurse is” (nurse, F3, S1).

#### 3.2.2. Subtheme 2: Generational Changes over Time Lead to Shifts in the Climate and the Nurses’ Rights in Ethical Decision-Making

In contrast to all other subgroups, the nurses observed that the climate was not “fixed”, but rather shifted and changed with the newer, younger generations of staff. This was first reflected in the relationships among the team members—and consequently, the ethical climate at their departments. However, there was no consensus on whether the impact of these generational changes was positive or negative. Some nurses opined that “there was no respect for the hierarchy anymore […] that’s what is being lost nowadays” (nurse, F3, S1)—a stance where they insinuated that their subordinate position within the medical team was something normal or that the extensive workloads were something to be expected. Others countered this, stressing that this brought about positive changes, as they were now more able to speak their minds on any moral or ethical dilemmas (Q11). Others, however, noted declines in patient–nurse relationships over time, where patients were behaving towards nurses in an openly hostile manner, while the younger generations of nurses were treating patients with less care and dedication (Q12). These changes, however, seemed to be a natural occurrence in the profession that came with age and experience, with one participant noting that she “has different stances towards everyone compared to when she was a younger nurse” (nurse, F2, S4) (Table 3).

While they could not agree on their merit, these nurses noted that these shifts in the ethical climate were inevitable, testifying to its fluidity and its independence from fixed rules and regulations. While some ethical and professional codes remain the same, behavioural norms and practices often change with the passing of time, as do ways in which individuals and teams operate when tackling ethical challenges.

### 3.3. Theme 3: The Institutional Leadership Can Mobilise Underutilised Tools and Bodies to Improve the Ethical Climate in the Hospital

Participants across groups observed significant gaps in the way the Hospital exercised its authority over the ethical climate. Central to this was the poor management of human resources; as they were overworked, staff members had little time to dedicate to both patients and colleagues, which reflected negatively on the ethical climate at the institutional and departmental levels. This was exacerbated by a lack of top-down support for resolving ethical dilemmas, with individuals feeling they were left “on their own” when struggling with conflicts with colleagues, patients, or the media. The Hospital leadership likewise failed to exercise punitive measures for ethical misdemeanors and malpractice, or to use the tools at its disposal to regulate the ethical climate within the institution, and mainly depended on staff members to do this themselves. Despite this, the staff still desired for the institution to be actively included in forming the ethical climate through these tools and bodies, as it would both improve trust and provide them with a clearer framework in which to operate.

#### 3.3.1. Subtheme 1: Experiences of Institutional Miscare and Mismanagement of Human Resources Lead to Bottom-Up Mistrust

All participants reported feeling overburdened by care-related obligations, documentation, and other tasks, which they attributed to a general lack of staff and the Hospital’s mismanagement of workflows and operating schedules. This consequently had an impact on the ethical climate within departments, as “being overburdened with work led to conflicts among staff” (nurse, F1, S2). Specialists, however, had differing opinions; while some noted that being overworked led to greater stress and “consequently, poorer communication with colleagues” (specialist, F6, S4), others claimed it does not, as “staying longer work is something the staff accept as normal” (specialist, F5, S3). Residents, meanwhile, generally did not feel overburdened “at the department, but did at the emergency ward” (resident, F4, S6), where they were under pressure to make ethically dubious decisions without oversight or advice from older colleagues (Q13). More importantly, this mismanagement of human resources and their workload meant that the Hospital staff had inadequate time and energy to dedicate to patients—the end users of their services (Q14). While our participants saw this as an ethical problem from a professional standpoint, they also perceived it to be deeply troubling at a personal level. This also led to dissatisfaction among patients, worsening their relationships with the staff. All these factors led the staff to perceive that the Hospital leadership did not care for the ethical climate, as it “did not want to tackle serious issues, and mainly dealt with trivialities” (nurse, F2, S1) and expected staff “to deal with everything themselves, or with their direct superior only” (specialist, F5, S2), or to “consult all relevant services and just get things done” (resident, F4, S3).

This “perceived miscare” was best exemplified in discussions on controversial cases publicised in the media or (physical or legal) pressures from/confrontations with patients, where most staff believed they would find no protection or help from the institution. This caused dissatisfaction among staff, as well as perceptions of a “negative” ethical climate and of worsening relations with patients (Q15). In fact, it seemed that the media and public opinion had a significant impact on the climate in the Hospital, especially the daily continuum of care and relationships between the healthcare staff and the patients (Q16). For example, when someone died within the Hospital, the staff automatically “turned towards more defensive medicine in treatment” (resident, F4, S1). There were different perceptions, however, about how much intra-team relationships were affected. Some reported that “[staff members] stick together and protect all colleagues” when faced with publicised cases (nurse, F1, S2). Others noted that “there are always some divisions [in such cases], as some colleagues will stand by the person involved and others will not […] so there will always be some friction” (resident, F4, S2).

Internally, the Hospital leadership did little to sanction staff members going against the ethical climate and infringing on other people’s rights or ethical/professional codes (Q17). As an example, participants complained of colleagues who went on sick leave indefinitely without any true medical indications, leaving others to do their work. Others noted cases of infringements of patients’ confidentiality, where staff members “looked into the Hospital records of other staff members out of pure curiosity” (HEC, F7, S1) or “printed out a patient’s case and forwarded it to a third person” (HEC, F7, S4). The Hospital’s legal department and ethics committee seemed to have no power in regulating these cases or sanctioning staff members. As one participant put it, “Even if someone breaks the code, what are the sanctions?” (HEC, F7, S4).

On the whole, the Hospital staff in our focus groups generally perceived that the institution had no role in defining the ethical climate. They felt that not only were they left to tackle ethical challenges themselves, but that they also could not rely on institutional protection in these situations. They also believed that even in cases where one individual went against a “good” ethical climate (i.e., a climate based on a team having identical professional and personal values), the institution would not try to enact any form of punishment, nor support bodies that could exercise adequate sanctions.

#### 3.3.2. Subtheme 2: Perceived Need for Better Use of Advisory Bodies, Codes, Protocols, and Guidelines by the Hospital Leadership

Participants across groups had limited knowledge or awareness of the Hospital’s internal ethical codes, rules, guidelines, and regulations. Some explicitly noted that the “Hospital has no [code], it just has some codes of conduct (nurse, F3, S1). Others said that the institution did not provide them with protocols, but rather that they “developed them on [their] own, with each MD taking care of one topic […] based on European, global and other protocols (specialist, F5, S1). They, therefore, called for the development of protocols that would “be practical” and guide them in everyday cases, such as when they have to treat an elderly patient with a broken hip without his legal carer present. They noted that, at the time being, these cases were “not described anywhere, in any [institutional] guidelines […] which bothers us a lot” (specialist, F6, S2). Given the “defensive nature of medical practice these days” (specialist, F6, S2), such protocols would also help define responsibilities in such situations, offering ethical and legal protection to staff (Q18).

As mentioned before, the HEC, an existing body within the Hospital, did not have purview over infringements of ethical and professional codes. One of its members reported that they “exist mostly to protect the participants of scientific research” (HEC, F7, S1), i.e., provide ethical approval for research within the Hospital. The staff, therefore, believed that the duties and authorities of the HEC should be expanded, and that the HEC itself should include representatives from all subgroups within the Hospital so that cases of ethical infringement would be tackled without bias towards any individual. In a similar fashion, they called for the establishment of “psychological support that could be used by all staff” (resident, F4, S1) or a similar body within the Hospital, where individuals could anonymously seek help in general or with specific ethical challenges (Q19).

Lastly, the staff called for additional education focused on either communication skills or ethics. To be effective, these lectures and training had to be led by specialised professionals and had to be designed for smaller teams, rather than large groups or whole departments. This was postulated on the assumption that “communication is something that is learned and trained” (nurse, F3, S1) and that “people willing to communicate will resolve everything, and this will result in a good climate” (resident, F4, S5). Additionally, the professionals leading the training and the staff attending it needed to be reimbursed financially for their time, as they would not take it seriously otherwise (Q20) (Table 4).

In short, it seemed that, despite the institution’s ambivalence towards the ethical climate in the Hospital, the staff wanted it to be involved more directly through the development or utilisation of protocols, guidelines, and other support systems. While some participants expressed dubiousness about their effectiveness due to a lack of a sanctioning system, they mainly agreed that any such intervention from the institution’s leadership could impact the climate positively, as it would help affirm a “good climate” by holding “bad actors” who infringed upon it accountable for their misdemeanor.

## 4. Discussion

In this study, we deepened the findings of our survey by exploring the staff’s perception of the ethical climate at the University Hospital of Split, Croatia, and identifying some factors that impacted it either positively or negatively. Three themes emerged in our analysis. The first explained the simultaneous predominance of the “Company rules” and “Laws and professional codes” we observed in our survey as stemming from the institutions’ expectations, rather than any actual rules, being in alignment with the staff’s professional values. The second theme focused on nurses’ perception of their “subordinate” position within the team, and its implications on the climate. The last theme highlighted some shortcomings and opportunities in the Hospital’s handling of the ethical climate, as seen by its employees.

These qualitative findings contextualise the results of our survey in the same hospitals [22]. Namely, in view of the ethical climate theory, they explain the occurrence of the “Laws and professional codes” climate, which manifests at the cross-section of the “cosmopolitan” locus of analysis and the “principle” construct [6]. In the context of our study, we observed how the staff believe the Hospital mainly expects them to follow their own professional codes in ethical decision-making. This consequently explains the simultaneous predominance of the “Company rules” climate, which emerges at the cross-section of the “Local” locus and the “Principle” construct. As seen from our focus groups, the staff perceive the two climates to functionally be one the same; they rely on their own professional, external codes (“Laws and professional codes” climate), yet also believe that this is exactly how their organisation expects them to behave (“Company rules and procedures” climate). To this, we also note that the Hospital is a publicly funded institution strictly regulated by the Ministry of Health of Croatia. As Viđak et al. [20] have hypothesised in their study at the University of Split School of Medicine, it is highly possible that these two climates are harder to distinguish from one another in cases where internal rules are based on external codes.

Our observation that the staff rely primarily on their deeply embedded professional values, rather than the Hospital’s codes or rules, aligns with previous research. Some studies have indicated that, in the absence of organisational support, healthcare workers fall back on professional grounds, identities, and norms to guide their behaviour [27,28]. Others have shown that when ethical infrastructure, such as HECs, is weak or insufficiently visible, healthcare staff fall back on professional values or adapt to their local team’s microculture [29,30]. Our participants likewise reported that they routinely resolved ethical dilemmas within their team or in consultation with their senior colleagues, rather than by turning to institutional bodies. This indicates that formal institutional mechanisms may be perceived as less accessible or relevant to everyday clinical practice. This aligns with the findings of a qualitative study exploring the ethical climate at a hospital in New Zealand [31], where nurses similarly reported relying primarily on their immediate nursing team for emotional and ethical support, while perceiving hospital management as distant, unresponsive, or primarily driven by budgetary concerns. These nurses also felt neglected when voicing concerns about patient safety and staffing levels, with their concerns being reframed by institutional leadership as personal weaknesses, rather than systemic problems [31]. Furthermore, evidence from previous research suggests that organisations with perceived caring ethical climates, characterised by mutual concern and support, have a stronger positive impact on trust in supervisors and the leadership than rule-based climates [32], which may also explain why professionals in latter settings are less likely to turn to formal institutional bodies and instead rely more on their immediate team and collegial relationships for ethical guidance, as is the case in our study.

As observed elsewhere, teams compensate for gaps in their institution’s ethical guidance by developing their own informal norms, which subsequently impact the ethical climate at their workplace significantly [33]. While this approach is not inherently negative, as professional norms—particularly in medicine and nursing—often reflect deeply internalised commitments to patient welfare, dignity, and fairness [34], it does bear some limitations. First, relying solely on professional codes can lead to considerable variability in clinical work across units, because codes of medical ethics are not static and do not provide uniform guidance on how to act in different situations, but instead allow for different interpretations in different contexts [35]. It might also reinforce existing hierarchies, whereby ethical standards of others are shaped by implicit norms of more dominant professional groups, usually MDs [36]—a phenomenon observed in the context of nurses in our focus groups. Lastly, employees often feel confused when they lack clear, institutional ethical guidance in the face of morally challenging or legally ambiguous situations, which leads to moral distress [37]. To prevent this, different stakeholders should be engaged when institutional rules are being established or revised [16,38].

Our findings indicate that the ethical climate is not experienced uniformly by all healthcare professionals. The nurses in our focus groups were seemingly more exposed to and aware of ethical dilemmas, as they had a more intense, direct contact with patients and acted as their “bridge” to MDs. This aligns with a literature review that showed how nurses across countries commonly face ethical dilemmas related to end-of-life decisions, conflicts with physicians or families, patient privacy, and organisational constraints [39]. A related concern is nurses’ lack of independence and control in their work, as they often feel inferior within medical hierarchies, particularly when doctors ignore their recommendations or when patients’ family pressure them to give more care, which leads to moral distress [40,41]. In such settings, where ethical dilemmas discussions take place mainly within hierarchical team structures, professionals with less decision-making authority, particularly nurses and residents, may experience moral distress if they feel unable to act according to their own ethical judgment [42]. The nurses in our focus groups similarly reported feeling “subordinate” to MDs within their medical teams, noting that they were expected to assume tasks typically within the MDs’ scope of practice, while being held accountable for potential errors, which can indicate hierarchical imbalances that may contribute to moral distress and perceptions of organisational unfairness. However, in modern-day medical teams, maintaining nurses’ professional autonomy and ensuring their position is recognised by MDs is critical, as this empowers them to participate in collaborative decision-making and is directly related to their job satisfaction [43].

The nurses in our study also perceived that institutional leadership was less protective of their professional rights than of the rights of MDs, whom the patients tended to respect more. This seemingly reflected a power imbalance in healthcare organisations observed in other research, where MDs traditionally had greater authority and influence in decision-making [44]. Furthermore, it has been reported that nurses perceive inequalities in pay, access to resources, training opportunities, involvement in decision-making, and policy enforcement as forms of organisational injustice, attributing these disparities to medical dominance [45]. Such hierarchical dynamics negatively influence the respect and validation nurses receive from their institution and can limit their ability to act on their ethical concerns [41]. The nurses in our study also highlighted how the staff’s age plays a role in forming the ethical climate, as younger nurses seemed to be more comfortable in openly criticising unethical behaviour and raising concerns of ethical dilemmas. Some, meanwhile, felt that younger generations were less committed to the traditional professional hierarchy and less oriented toward ensuring the quality of patient care. This could be related to nurses’ tendency to follow the MDs’ moral principles and standards rather than their own, as the latter hold the highest position in the professional hierarchy. However, research among nurses from generation Y (born between 1980 and 2000) showed them to be more vocal, driven by strong altruistic values, and committed to their work when they feel aligned with and supported by their organisation [46]. However, they also expect their institution to be flexible and ensure their life-work balance, and are willing to leave their job when these needs are unmet, while resisting any form of rigid hierarchy that would infringe on their rights [46].

Relatedly, our participants also reported feeling mismanaged by the Hospital, where high workloads, understaffing, and inefficient organisational processes left them limited time for patients and colleagues, exacerbating interpersonal tensions and deteriorating the ethical climate. As a consequence, Hospital staff did not have enough time to inform patients about medical procedures and expected waiting times, which had a negative impact on the patient-healthcare professional relationship. The aforementioned qualitative study from New Zealand showed that top-down pressures related to patient flow and organisational targets not only generated moral tension for nurses, but also forced them to overlook professional and ethical commitments to patient care [31]. It has been shown that high workloads and understaffing among medical professionals may lead to mental fatigue, an increased incidence of medical errors, and a decreased quality of care for patients [47,48]. Conversely, high-performance human resource management systems which support staff development and engagement tend to be characterised by lower patient mortality and improved quality of care [49]. One study conducted in the Netherlands [50] found that human resource management improved a hospital’s financial results and patient and employee satisfaction and reduced the number of days employees spent on sick leave.

Our participants presented several examples of misconduct, such as inappropriate access to medical records and breaches of confidentiality, where the Hospital imposed little to no sanctions on the transgressors. This led them to lose confidence in the leadership’s ability or willingness to improve the ethical climate by tackling such cases of misbehaviour. This aligns with the findings of a qualitative study of hospital staff in Australia which reported that unprofessional behaviours and a lack of organisational response to such incidents can undermine trust in leadership and reinforce perceptions that the organisation is unwilling or unable to uphold ethical standards [51]. It has been shown that, in institutions where policies are poorly formulated and sanctions rarely imposed, staff often lose confidence in the leadership’s ability to address or prevent unprofessional conduct, undermining top-down efforts to improve the organisational culture or working conditions [51]. Similarly, our participants noted that the Hospital did not fully or appropriately use its formal authority and existing mechanisms and bodies to influence the ethical climate, suggesting a gap between formal hospital ethical structures and their practical implementation. Previous studies have demonstrated that, in modern organisations, ethical leadership that prioritises moral principles and values in decision making is essential for establishing ethical expectations, guiding employees in moral dilemmas, and ensuring fair practices for dealing with misconduct [52,53]. In the context of healthcare, research has shown that leadership that fosters awareness of ethics contributes to greater job satisfaction and stronger employee commitment to the institution, while also helping mitigate staff burnout [54].

Since circa 1997, Croatia’s Law on Health Protection mandates that all healthcare institutions should form HECs, with each including five members—three medical and two non-medical professionals. These HECs, formed through a top-down approach, are also legally obliged to review research protocols; this, in practice, led to confusion regarding their roles, since research-related tasks often take precedence over support in ethically dubious clinical cases [55]. The staff in our focus groups likewise felt that the role of the HEC was limited to approving scientific research within the Hospital only, rather than handling ethical dilemmas and helping sanction unethical behaviour. This was confirmed by the HEC members themselves, who reported having very limited authority to intervene in such cases. This negatively impacted the ethical climate, as the staff believed they had no one to turn to for help when facing ethical challenges, while the HEC felt dismayed because they could not assist the staff.

While countries in the Americas and Europe have well-functioning HECs, many Eastern Mediterranean and South-East Asian countries, usually of lower-income or transitional economic status, are only beginning to establish their own and continue to face basic challenges in defining their role, involving patients and families, and providing ethics education [56]. A qualitative study among 19 HEC members from Tehran, Iran, reported on HECs’ low executive power and inefficiency in hospitals’ policymaking [57]. Similar observations emerged from a ten-year study in Turkey, where the role of the HEC remained unrealised despite frequent ethical dilemmas [29]. The causes behind this were low awareness among staff members, concerns about professional autonomy, and doubts about the HEC’s efficacy, despite its recommendations contributing to policy development and highlighting a need for a more structured decision-making process in resolving ethical dilemmas. Conversely, the HEC of Stanford Hospital plays an active, comprehensive role in policy development and addressing ethical issues [58].

Although this study was conducted within a single Croatian hospital, several of its findings resonate with international research on ethical climate, moral distress, and hierarchical dynamics in healthcare organisations. Evidence from healthcare system in Eastern Europe suggests that healthcare professionals frequently encounter ethically stressful situations related to organisational constraints, hierarchical relationships, and challenging interpersonal dynamics in clinical settings, indicating that unfavourable ethical climates may be more common in healthcare systems with strong professional hierarchies, limited resources, and underdeveloped institutional ethics frameworks [59]. Our study adds to this knowledge by exploring these common phenomena in a healthcare system where formal ethical frameworks and institutional bodies are still under development. Furthermore, studies examining post-socialist healthcare systems in Central and Eastern Europe highlight that structural and socioeconomic constraints, such as limited resources, unequal access to healthcare services, and organisational challenges continue to shape healthcare delivery and working conditions in the region [60,61]. Our findings may thus be relevant to other organisational and national contexts that face similar challenges in integrating institutional ethics policies into routine clinical practice for the first time. Such institutions and their leaderships could, for example, consider providing staff with practical protocols related to cases they encounter in practice, rather than generalised normative guidance. They could also develop institutional bodies that would support staff through psychological consultations, while ensuring their anonymity in the process. Lastly, any institutional bodies tasked with shaping the ethical climate in an institution should have the legal power to sanction unwanted behaviour. This would not only define what is desirable in the context of the ethical climate, but also provide staff with a feeling that they would be cared for by their institutions in cases of moral distress caused by patients or colleagues.

Our focus group has several strengths. First, we included healthcare professionals from various subgroups within the Hospital, allowing us to explore the perspectives of individuals from across the institutional hierarchy. We also introduced bias checks, where an unbiased observer participated in the focus groups and later conducted the coding in parallel with the moderator and main coder, who knew some of the focus group members professionally. However, some limitations should be noted. Given the relatively small size of the study site, three of our researchers (ZH, JK, LjZ) knew some of the focus group members personally, through professional contacts. The roles of the latter two (JK, LjZ) within the Hospital hierarchy (member of Department of Health Care Quality and head of the HEC, respectively) may have created perceived power dynamics or expectations that could have influenced participants’ willingness to speak openly, leading to social desirability bias. Furthermore, we conducted this study in a single setting only; insights from staff at other hospitals in Croatia might differ from ours, as might the findings of research in other countries and contexts. Lastly, we were unable to capture the perspectives of staff across all departments or with varying years of experience and as gathering the already overstretched staff across departments and shift proved difficult. However, we note that exploring such nuances was outside of the scope of our study, as it was based on existing theoretical and empirical data and sought to explore how two pre-identified normative climates manifested among the staff at the Hospital.

Our findings provide several possible approaches for improving the ethical climate for management of hospitals in Croatia or similar contexts, where the challenges observed in our focus groups might also emerge. First, irrespective of external codes followed by healthcare staff, healthcare institutions should provide rules, guidelines, and codes of their own. These can themselves be grounded in the healthcare professionals’ values and codes, but they need to provide clear, practical protocols for tackling everyday ethical dilemmas, such as issues of insufficient hospital beds, saturated work schedules, and so on. Second, hospital administrations should strive to set up or capitalise on established institutional bodies and tools for regulating the ethical climate, such as HECs, anonymous psychological or legal consultations, and ethics hotlines, allowing them wider visibility and berth in exercising their authorities. Third, a special support system that would alleviate their feelings of subordination to MDs and their exposure to patients and the media needs to be placed for nurses. While this could be partially resolved through dedicated bodies within hospitals, their administrations also need to take an active, visible role in managing the within-team hierarchies and in standing behind their employees during times of crisis. Furthermore, they should also use punitive measures, where appropriate, to sanction misbehaviour that would infringe on the ethical climate. Lastly, improving the ethical climate can be considered an investment in healthcare quality and organisational resilience. By promoting open communication, consistent accountability, and mutual respect across professional groups, healthcare institutions can improve care coordination, support staff engagement, reduce burnout and turnover, and ultimately enhance patient safety and overall effectiveness of care delivery.

## 5. Conclusions

While our survey data show the simultaneous predominance of the “Laws and professional codes” and “Rules” climates, our qualitative findings suggest that staff primarily define and navigate the ethical climate on their own according to their professional values. The Hospital itself sets no explicit rules for them to follow, but rather inadvertently expects them to behave as professionals, i.e., by their professional codes. This leads to differences in how the ethical climate manifests across professional subgroups, with nurses being especially sensitive to its shifts as “frontline workers”. Due to these reasons, the staff call upon the Hospital administration to step in and utilise the tools and bodies at its disposal to define the ethical climate and explicitly regulate how staff should act when faced with ethical dilemmas, both among themselves and in patient care.

## Figures and Tables

**Table 1:** Characteristics of focus group participant.

	FG1—Nurses (n = 4)	FG2—Nurses (n = 5)	FG3—Nurses (n = 5)	FG4—Residents (n = 6)	FG5—Specialists (n = 3)	FG6—Specialists (n = 4)	FG7—Hospital Ethics Committee (n = 4)
**Sex**							
Men	1	1		3	-	3	
Women	3	4	5	3	3	1	4
**Level of education**							
Nursing high school	-	-	1				
Bachelor’s degree in nursing	2	2	3				
Master’s degree in nursing	2	3	1				
Medical doctor				6	3	4	2
Lawyer							2

FG—focus group.

**Table 2:** Quotes related to the subthemes of theme 1: The Ethical Climate Is Formed by People and Teams Based on Shared Professional Values.

Quote	Participant
Q1: Some rules need to exist in an institution like this and in a job like ours, and we all need to follow them. No one thinks these things up by themselves—what we do is what we were taught, and we all must respect this and function in this way.	Specialist, F5, S1
Q2: [We learn them] at university, through most of our basic education at university. You have the Hippocratic Oath, everything you’re taught by lectures and the professors who transfer their knowledge and behaviour. These rules transferred [to our work].	Specialist, F5, S1
Q3: The climate at the department is very good […] We don’t have any formal guidelines, nor did we ever get them. It’s actually somehow intrinsically related to our collective.	Nurse, F4, S3
Q4: We always try to resolve things between ourselves. If a nurse has done something, we sort it out within the team. If that doesn’t work, we go one step higher, and then another. We haven’t yet had to involve the institution.	Nurse, F3, S4
Q5: We’re the junior staff. I think it’s best that we communicate everything with the seniors, as they’re the ones who are responsible for everything in the end.	Resident, F4, S5
Q6: If you are a specialist, you’re ethically responsible for your own actions. Someone can advise you more or less, but it’s all up to you in the end; your boss can tell you what to do, but you always act how you think is morally right.	Specialist, F6, S1

**Table 3:** Quotes related to the subthemes of theme 2: As “Frontline Workers”, Nurses Are the Most Sensitive to the Ethical Climate and Its Shifts.

Quote	Participant
Q7: We nurses do a lot of things that MDs should […]. Let’s start with transfusion—does the MD do it or the nurse? We decided that the MD should, but this isn’t how it’s done in practice.	Nurse, F1, S3
Q8: Those [ethically problematic] cases are not in the nurses’ professional purview, but we are forced to be present when someone decides on continuing the treatment or not. This makes me feel really uneasy.	Nurse, F2, S4
Q9: Nurses are always strung up in the press when there’s a problem, which isn’t the case with doctors, who are often “not named” [anonymous] in these cases. I haven’t experienced this, but I don’t feel that I would be protected [by the institution] if this would ever happen.	Nurse, F1, S2
Q10: You have to remain cool, even if it makes you crazy. The worst thing is that I know the patient would calm down when you call the MD back. That makes me mad.	Nurse, F1, S4
Q11: Times have changed—you can more freely express yourself now. The generations of MDs changed, too. Before, you had to keep quiet, as we were just “little nurses”—we worked and could never say anything […]. This is changing now—you can speak your mind; there’s some pushback, but I can do this now, and no one can stop me.	Nurse, F3, S5
Q12: Our relationship with the patients changed. I’ve been in the Hospital for 15 years and can see this. You used to respect them more, but they also behaved differently towards us. This all got lost somehow.	Nurse, F2, S2

**Table 4:** Quotes related to the subthemes of theme 3: The Institutional Leadership Can Mobilise Underutilised Tools and Bodies to Improve the Ethical Climate in the Hospital.

Quote	Participant
Q13: It’s about the amount of work to do and the number of staff. This can also create a good or bad ethical climate. If work has been poorly managed by superiors, this transfers to the employees, so some of them might not follow or know the rules, or respect authority.	Nurse, F2, S4
Q14: We can’t inform patients. If our normative is to check 12 or 18 patients in an eight-hour shift, where you have six to seven hours of effective work, we can’t manage 47 patients as we do now. We can’t dedicate ourselves or inform them as we’d want. You’re limited; you can print out the paper and just quickly explain things to him, but he usually remains uninformed, which generates everything else [all other ethical issues].	Specialist, F6, S4
Q15: The institution questions us for the smallest things, such as complaints from people in the waiting room […]. When you read some for those e-mails, you clearly see they are unfounded, but you still have to respond and explain yourself. This is really denigrating.	Nurses, F3, S5
Q16: The media are extremely powerful, and people are afraid. The press are unprofessional, and their info is usually incorrect or unverified. We had the Legionella outbreak story now […] they affect our work and care too much, but also our relationships with our colleagues. When something happens, people want to shift the blame.	Specialist, F6, S3
Q17: I balance between the visits, the MDs, the patients, my colleagues […] but there is no system of punishment for non-ethical behaviour. This creates a poor climate we are personally responsible for, but the institution should find a way to regulate this. The problems are always cause by the same people.	Nurse, F3, S4
Q18: We need the ethical code to focus on the things we encounter the most, but are the least interesting. What if we have a patient from the [nearby island] who has to stay for a longer time in the Hospital, and he can’t come back later? […] What if the waiting list is too long? We need it to tackle cases where you can’t do your job properly.	Specialist, F6, S4
Q19: It’d be really useful to have some [psychological or ethical] support for these cases, but this should be anonymous, so that you’d have a person who is neutral.	Specialist, F5, S1
Q20: We need lectures on ethics, but paid ones. We can’t expect the employees to do something themselves in their free time. They can stay longer at work and listen to a lecture, but it needs to be paid—this, and only this will produce some results.	Specialist, F6, S4

## Data Availability

As the focus group transcripts contain sensitive information that could reveal the participants’ identities, the ethical committee did not approve of their sharing.

## Supplementary Materials

The following supporting information can be downloaded at: https://www.mdpi.com/article/10.3390/healthcare14060735/s1, File S1: Topic guide: Focus Group Guide for Nurses/Residents/Specialists, Figure S1: Themes and subthemes identified in the analysis; Table S1: COREQ checklist.

## Abbreviations

The following abbreviations are used in this manuscript:

HEC	Hospital Ethics Committee
COREQ	Consolidated Criteria for Reporting Qualitative Research
