# Supplementary material for: Perception of the Ethical Climate Among Hospital Employees in a Public Healthcare System: A Qualitative Study at the University Hospital of Split, Croatia
Source: Healthcare (Basel). 2026 Mar 13;14(6):735. doi: 10.3390/healthcare14060735 (PMC13026490; doi:10.3390/healthcare14060735)
Supplement: Supplementary file 1 [file healthcare-14-00735-s001.zip › Supplementary File S1.pdf]

FOCUS GROUP GUIDE FOR NURSES/RESIDENTS/SPECIALISTS

Introduction

[INTRODUCTION OF THE MODERATOR/FOCUS GROUP OBSERVER]

This focus group is being conducted as a follow up of the research study "Perception of the Ethical Climate at the University Hospital of Split: A Cross-Sectional Study." Everything you say here will remain anonymous; only researchers within our working group will have access to the recordings and transcripts. Furthermore, the transcripts will be de-identified during analysis. Individual statements will be translated into English for the purposes of scientific publication and included in a scientific article. They will not contain any information that could reveal the identity of the participants.

Please note that you may withdraw your consent at any time until the end of the focus group.

Introduction to the concept of ethical climate and the findings of the first cross-sectional study

The concept of ethical climate refers to the atmosphere prevailing within an organization in terms of moral principles, integrity, and employee behavior. It encompasses values, rules, norms, perceptions, and expectations that influence how people behave and make decisions in their everyday work environment.

Prompt 1. Based on this definition, how would you describe the ethical climate in your workplace?

Prompt 1.1. How is the ethical climate manifested in your departments – does it stem from you personally, from your colleagues, or from certain institutional expectations?
Prompt 1.2. How do you align your personal values with the ethical expectations and ethical climate in your work environment? Do you see their influence in your daily work?
Prompt 1.3. How do you approach decision-making in your workplace when faced with a morally ambiguous situation? Do you resolve such situations independently, with colleagues, or in some other way?
Prompt 1.4. How do you think you are expected to act in ethically questionable situations, whether involving colleagues or patients?

Prompt 2. How does your institution influence your work environment?

Prompt 2.1. Our findings show that the two dominant ethical climates at the University Hospital of Split are "institutional rules and procedures" and "laws and professional codes." Both are associated with greater organizational commitment and positively influence employee job satisfaction. They also suggest that employees are primarily guided by "external" expectations and rules. What are your thoughts on this?

Prompt 2.2. Are you automatically expected to work more than is legally required? Do you consider yourself overburdened with work? How is this reflected in the ethical climate in your context?

Prompt 2.3. To what extent do you believe ethical principles are integrated into decision-making processes in the hospital, in terms of treatment of employees and patients? Is this reflected in your department?

Prompt 3. Who (or what) has the greatest influence on the ethical climate within the institution?

Prompt 3.1. Regarding the ethical climate, do you believe the institution has responsibility and influence toward you as employees? Do you believe it is willing to protect you in "ethically questionable" situations?

Prompt 3.2. What is your institution's social responsibility? Do situations in the public sphere/media/real life influence the ethical climate in your department or hospital in general?

Prompt 3.3. What changes or improvements would you propose to enhance the ethical climate at the University Hospital Centre Split in the contexts previously mentioned?

Prompt 4. Introduction to the concept of a code of ethics – rules of conduct for employees and the ethical principles upon which employees act in the performance of their duties

Prompt 4.1. Currently, there is no formal hospital code of ethics; only the professional chamber's code is referenced. Are you familiar with it?

Prompt 4.2. Do you believe your institution should invest in developing clear ethical guidelines and provide support to employees through procedures for reporting irregularities? Would this affect the climate and help in ethically questionable situations?

Prompt 4.3. Can you provide examples in which a code of ethics would be important to you?

Prompt 4.4. What rules/guidelines would you include in your institution's Code of Ethics?

ADDITIONAL QUESTIONS FOR HOSPITAL ETHICS COMMITTEE

Prompt 5. How does your institution influence your work environment?

Prompt 5.1. What steps does the hospital take to educate its staff about ethical standards and practices?

Prompt 5.2. Is there regular training or professional development programs related to ethical issues for medical staff? If not, do you think this would help employees?

Prompt 5.3. How are information about ethical standards and procedures communicated to hospital employees?

Prompt 5.4. To what extent are hospital employees aware of the existence of the Ethics Committee and its activities? Likewise, of the Legal Department?

Prompt 5.5. What role does the Ethics Committee play in providing advice and support to hospital staff?

Prompt 5.6. How does the hospital deal with reported ethical problems or incidents?

Prompt 5.7. What procedures and sanctions are predicted in cases of violations of ethical standards?
Prompt 5.8. Can you provide an example of how the hospital handled a specific case of an ethical breach?
Prompt 5.9. In what way does the hospital ensure confidentiality and protection for those who report ethical concerns?

Prompt 6. Who (or what) has the greatest influence on the ethical climate within the institution?

Prompt 6.1. Regarding the ethical climate, do you believe the institution has responsibility and influence toward its employees? Do you believe it is willing to protect them in "ethically questionable" situations? Do employees seek protection from the Ethics Committee or the Legal Department?
Prompt 6.2. Do situations in the public sphere/media/real life influence the ethical climate in the hospital overall? Have there been situations where employees asked the hospital to protect them in dealings with the media?
Prompt 6.3. What changes or improvements would you propose to enhance the ethical climate at the University Hospital of Split in the previously mentioned contexts?

Prompt 7. Introduction to the concept of a code of ethics – rules of conduct for employees and the ethical principles upon which employees act in the performance of their duties

Prompt 7.1. Currently, there is no formal hospital Code of Ethics; only the professional chamber's code is referenced. Who is involved in drafting the Code of Ethics?
Prompt 7.2. What principles guide you in drafting the Code of Ethics, and which guidelines do you intend to include in it? Would this influence the climate and help in ethically questionable situations?
Prompt 7.3. How do you think it can be ensured that all employees are familiar with the provisions of the Code? Will you take employees' suggestions into account when drafting the Code?
Prompt 7.4. Can you provide examples in which a Code of Ethics would be important for healthcare professionals at the University Hospital of Split?
