# Supplementary figures and images for: Perception of the Ethical Climate Among Hospital Employees in a Public Healthcare System: A Qualitative Study at the University Hospital of Split, Croatia
Source: Healthcare (Basel). 2026 Mar 13;14(6):735. doi: 10.3390/healthcare14060735 (PMC13026490; doi:10.3390/healthcare14060735)

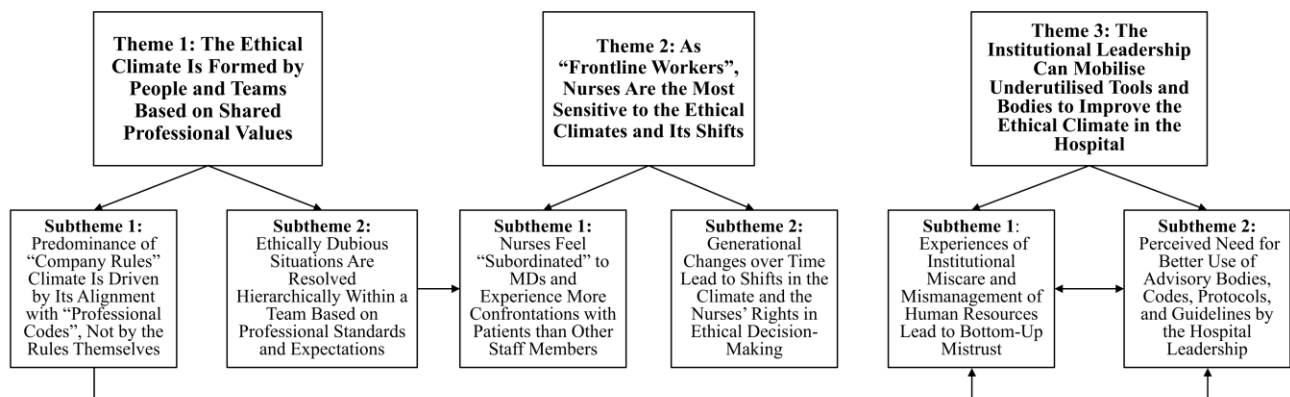

**Figure S1.** Themes and subthemes identified in the analysis.

Supplement: Supplementary file 1 [file healthcare-14-00735-s001.zip › Supplementary Figure S1.pdf]
